# Ubrogepant, erenumab, and eptinezumab antagonize positive inotropic effects of the calcitonin gene–related peptide in the isolated human atrium

**DOI:** 10.1007/s00210-025-04029-7

**Published:** 2025-03-14

**Authors:** Joachim Neumann, Britt Hofmann, Ulrich Gergs

**Affiliations:** 1 Institute for Pharmacology and Toxicology, Medical Faculty, Martin Luther University Halle-Wittenberg, Magdeburger Straße 4, Halle, Saale D-06112 Germany; 2 Department of Cardiac Surgery, mid-German Heart Center, University Hospital Halle, Ernst-Grube-Straße 40, Halle, Saale D-06097 Germany

**Keywords:** CGRP, CGRP receptor, Human atrium, Migraine therapy

## Abstract

The calcitonin gene–related peptide (CGRP) is an endogenous peptide that is known to be involved in the development of a migraine. CGRP is also present in the human heart, acts via CGRP receptors, and has been shown to increase the force of contraction (FOC) in isolated, electrically driven human atrial preparations (HAP) from adult patients obtained during open-heart surgery. Here, the hypothesis was tested that the positive inotropic effect (PIE) of CGRP could be attenuated by three anti-migraine drugs, namely ubrogepant, erenumab (both CGRP receptor antagonists), and eptinezumab (a CGRP antagonist). CGRP, cumulatively applied at concentrations ranging from 1 to 100 nM, increased the FOC. In the presence of cilostamide, an inhibitor of phosphodiesterase III, CGRP was more potent and effective than in the absence of cilostamide. Furthermore, when 100 nM CGRP was administered, subsequent application of ubrogepant (1 nM), erenumab (2 nM), and eptinezumab (6 nM) led to a reduction of FOC in HAP. In a more effective way, 1 µM carbachol and 1 µM (-)-N^6^-phenylisopropyladenosine (PIA) attenuated the PIE of CGRP in the presence of cilostamide. Conversely, when we applied first ubrogepant (1 nM), erenumab (2 nM), or eptinezumab (6 nM), then, this pre-incubation attenuated the PIE in HAP of cumulatively applied CGRP compared to CGRP given alone. We conclude that ubrogepant, erenumab, and eptinezumab are functional antagonists of CGRP in HAP at therapeutic concentrations of these anti-migraine drugs. Further investigation is necessary to determine whether this reduction in FOC is beneficial or detrimental for migraine patients.

## Introduction

Globally, migraine has a prevalence of approx. 15% and is known to affect a variety of areas of daily life. It is assumed that migraines are caused by the interaction of genetic and environmental factors. For instance, it has been demonstrated that the activation of the trigeminovascular system, leading to the release of vasoactive neuromodulators, including the calcitonin gene-related peptide (CGRP), amylin, the pituitary adenylate cyclase activating polypeptide (PACAP), and nitric oxide (NO), is involved in the pathophysiology of migraine and its progression to chronic migraine (Raggi et al. 2024). The objective of acute treatment of a migraine attack is to alleviate the symptoms that occur. In contrast, the pharmacotherapy to prevent migraine attacks aims to reduce the frequency and severity of these attacks. In the past, pharmacological treatment of migraine has been largely confined to triptans and non-steroidal anti-inflammatory drugs, as well as various oral preventive medications originally developed for other conditions, such as antihypertensives, antidepressants, and antiepileptics (Pellesi et al. 2024). Recent therapeutic advances have demonstrated the efficacy of targeted interventions in the CGRP signaling pathway in the treatment of migraine attacks. These therapeutic strategies, which include monoclonal antibodies and small molecules known as gepants, have demonstrated remarkable efficacy in clinical trials and offer a novel approach to the treatment of migraine (Pellesi et al. 2024). Here, we were interested in the cardiac effects of CGRP and possible cardiac side effects of drugs targeting the CGRP signaling pathway.

Human α-CGRP is a peptide composed of 37 amino acids and is formed in the human body in various organs, including the heart. The same gene (calcitonin gene, CALCA gene) leads by alternative splicing to calcitonin formation in thyroid cells and to the formation of α-CGRP in other cell types like the human heart (Rosenfeld et al. 1981; Russo and Hay 2023). Indeed, α-CGRP has been demonstrated to exert a positive inotropic effect (PIE) in human atrial preparations (HAP) (Franco-Cereceda et al. 1987a; b). Furthermore, when CGRP was intravenously injected into patients, it induced hypotension, vasodilatation, and elevated cardiac ventricular contractility (Franco-Cereceda et al. 1987a; Gennari et al. 1990). The physiological role of CGRP in the human heart is still incompletely understood and therefore remains speculative and enigmatic. One hypothesis suggests that locally produced CGRP or plasma CGRP can stimulate cardiac CGRP receptors, which exhibit a high affinity for CGRP (Russo and Hay 2023).

**Fig. 1:**
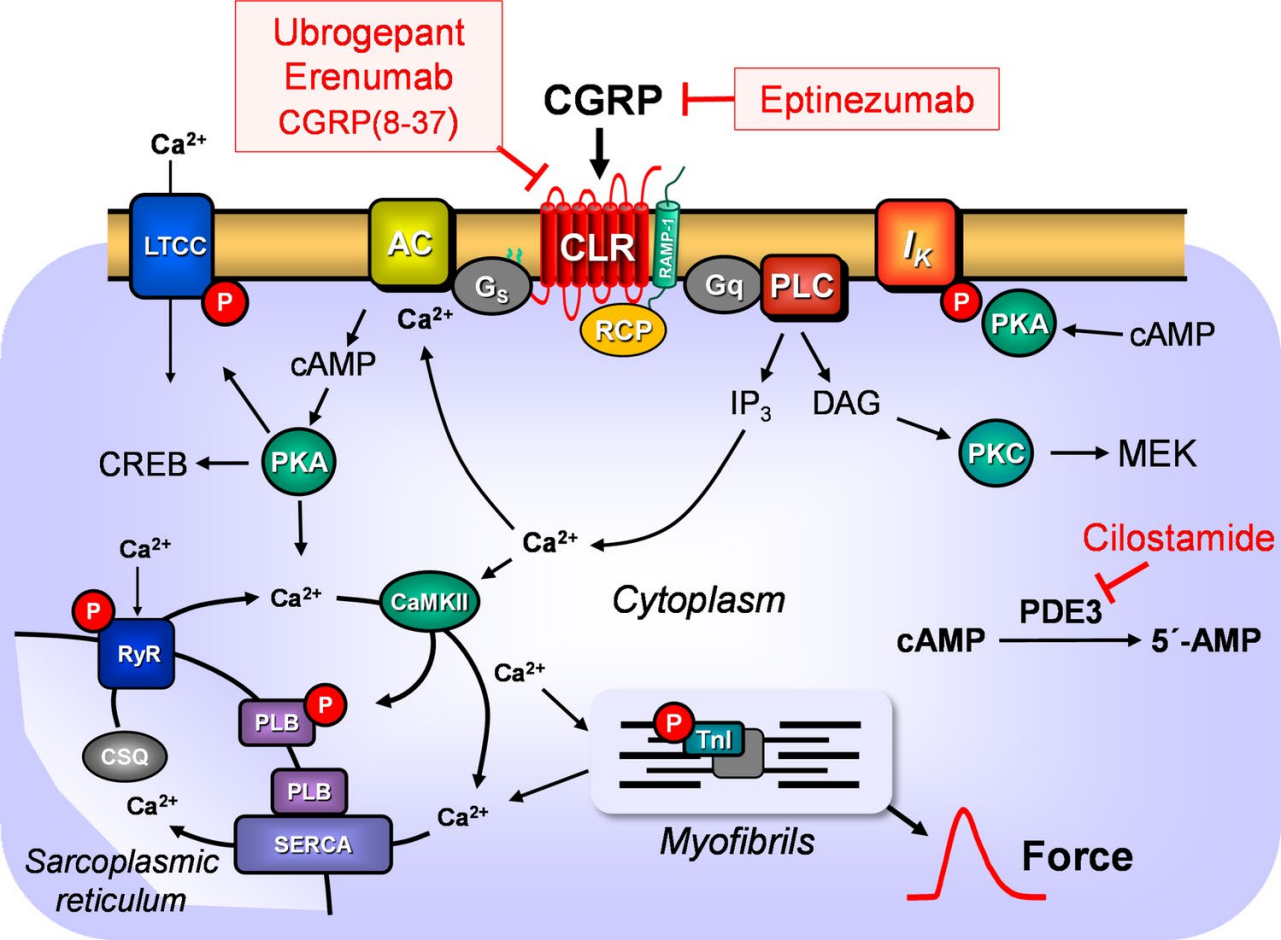
Schematic illustration of the mechanism of action of CGRP. Putative mechanism(s) of action of CGRP in cardiomyocytes. CGRP stimulates the CGRP receptor complex (CLR, calcitonin receptor-like receptor; RAMP1, receptor activity–modifying protein 1; RCP, receptor component protein). CGRP(8–37), ubrogepant, and erenumab are CGRP receptor antagonists, whereas eptinezumab is a CGRP antagonist. The CGRP receptor can activate two signaling pathways. Via stimulatory GTP-binding proteins (G_s_) and adenylyl cyclases (AC), the formation of cAMP is catalyzed. This cAMP activates a cAMP-dependent protein kinase (PKA). Subsequently, PKA regulates cardiac proteins through phosphorylation (P), such as phospholamban (PLB), the inhibitory subunit of troponin (TnI), the L-type Ca^2+^ channel (LTCC), potassium channels (*I*_K_), the ryanodine receptor (RyR), and the cAMP responsive element binding protein (CREB), which activates genes in the nucleus of the cardiomyocyte. Finally, the cAMP is degraded by phosphodiesterases (mainly PDE 3), which can be partially inhibited by cilostamide in the human heart. The CGRP receptor can also activate phospholipase C (PLC) via G_q_ proteins, which generates inositol trisphosphate (IP_3_) and diacylglycerol (DAG). DAG activates protein kinase C (PKC), which phosphorylates, for example, the mitogen-activated protein kinase (MEK). Myofibrils are responsible, in a Ca^2+^-dependent manner, for the generation of force, which is symbolized here by a single muscle contraction tracing over time. Ca^2+^ also activates the Ca^2+^ calmodulin-dependent protein kinase II (CAMKII). The contraction cycle is terminated by the sarcoendoplasmic Ca^2+^-ATPase (SERCA), which pumps the Ca^2+^ into the sarcoplasmic reticulum, where it is bound to calsequestrin (CSQ)

As concerns the mechanism of action (Fig. 1), CGRP can stimulate the activity of adenylyl cyclases (AC) in the heart and thus raise cAMP like isoprenaline (Sigrist et al. 1986). CGRP elevated the force of contraction (FOC) in cardiac preparations from guinea pigs and rats (Sigrist et al. 1986). For instance, in rats, these effects are region-dependent: CGRP increased the FOC in the left atrium but not in the ventricle of the rat (Sigrist et al. 1986). In spontaneously beating guinea-pig right atrium, CGRP increased the beating rate (Franco-Cereceda and Lundberg 1985). In addition, CGRP has been demonstrated to stimulate phospholipase C G_q/11_-dependently via its receptor, resulting in Ca^2^⁺ release from the endoplasmic reticulum (Drissi et al. 1998).

Two relevant receptors have been identified to which CGRP binds: the calcitonin receptor-like receptor and the calcitonin receptor. Both receptors can form complexes with distinct proteins called RAMP (receptor activity modifying proteins) (Russo and Hay 2023). In humans, the affinity of CGRP for complexes containing the calcitonin receptor–like receptor and RAMP1 are highest and thus might have the main clinical relevance and thus will only be considered further (Russo and Hay 2023).

The injection of CGRP has been demonstrated to induce migraine (Russo and Hay 2023). Consequently, CGRP antagonist are currently used as a therapeutic modality in the management of migraine (Russo and Hay 2023). Eptinezumab, an antibody, can bind and block the action of CGRP (Dhillon 2020; Li et al. 2023). Ubrogepant, a small organic molecule, blocks the CGRP receptor and has been approved for the prophylaxis and acute treatment of migraine (Boinpally and Lu 2022). Other anti-migraine medications are antibodies that inhibit the function of the CGRP receptor. One example is erenumab (= AMG 334) that we decided to study here (Shi et al. 2016; Vu et al. 2017; Markham 2018; Russo and Hay 2023).

Therefore, the primary objective of this study was to test the following hypotheses:

1. Does ubrogepant reduce the PIE of CGRP in HAP?
2. Does erenumab reduce the PIE of CGRP in HAP?
3. Does eptinezumab reduce the PIE of CGRP in HAP?

## Materials and methods

### Contractile studies on human atrial preparations

The contractile studies on HAP were performed using the same setup and modified Tyrode’s solution as described before (Gergs et al. 2009; Gergs et al. 2014). In brief, human right atrial preparations obtained during the cardiac surgery at the sites where extracorporeal circulation needles were inserted, were rapidly transferred into the laboratory in modified Tyrode’s solution. The modified Tyrode’s solution contained in millimolar concentrations (mM): 119.8 NaCI, 5.4 KCI, 1.8 CaCl_2_, 1.05 MgCl_2_, 0.42 NaH_2_PO_4_, 22.6 NaHCO_3_, 0.05 Na_2_EDTA, 0.28 ascorbic acid, and 5.05 glucose. Ascorbic acid was used as an antioxidant to maintain the activity of, for instance, isoprenaline. The solution was continuously gassed with 95% O_2_ and 5% CO_2_ and maintained at 37 °C and pH 7.4. The atrial samples were cut into small trabecular muscle pieces. These muscle strips were then mounted under isometric conditions with metal hooks at each end of the muscle in a glass organ bath. The muscle strips were electrically stimulated at 1 Hz with rectangular impulses of 5 ms duration and 10% above the voltage required for initiation of the beating (around 10 Volts). Human muscle strips were stretched to the maximum of the force-contraction relationship. The signals from the force transducers were recorded using a PowerLab system together with the software Lab Chart 8.0 (ADInstruments, Oxford, UK). The HAP were obtained from 28 patients, aged from 57 to 82 years (mean age ± SD: 68.7 ± 8.1 years). The patients suffered from severe coronary heart diseases (two- and three-vessel diseases). The cardiac drug therapy included acetylsalicylic acid, a factor Xa inhibitor, a diuretic drug and a β-adrenoceptor antagonist. Cardiac comorbidities included in part also hypertension and heart failure. As described in the figure legends, CGRP was applied in a cumulative manner, either alone or after application of cilostamide. In some experiments, the anti-migraine drugs were applied finally. In other experiments, the anti-migraine drugs were applied first, and then, CGRP was added cumulatively to the organ baths.

### Data analysis

The data presented herein are expressed as means ± standard deviation. The statistical significance was estimated using the one-way or two-way analysis of variance (ANOVA) as appropriate, followed by a Bonferroni multiple comparisons test. The statistical analysis and generation of graphs were carried out with the software Prism 9.0 (GraphPad Software, Boston, MA, USA). A *p* value less than 0.05 was considered to be statistically significant. The statistical power was calculated with G*Power 3.1.9.2. (Faul et al. 2009).

### Drugs and materials

(-)Isoprenaline tartrate, carbachol and (-)-N^6^-phenylisopropyladenosine were from Sigma-Aldrich (Taufkirchen, Germany). Erenumab was from Sellekchem (Köln, Germany). CGRP, eptinezumab and ubrogepant were from Thermo Fisher Scientific (Bremen, Germany) or Hycultec (Beutelbach, Germany), respectively. Human calcitonin was obtained from (Bachem, Bubendorf, Switzerland). All other chemicals were of the highest purity grade commercially available. Deionized water was used throughout the experiments to prepare the modified Tyrode’s solution. Stock solutions were prepared fresh daily.

## Results

**Fig. 2:**
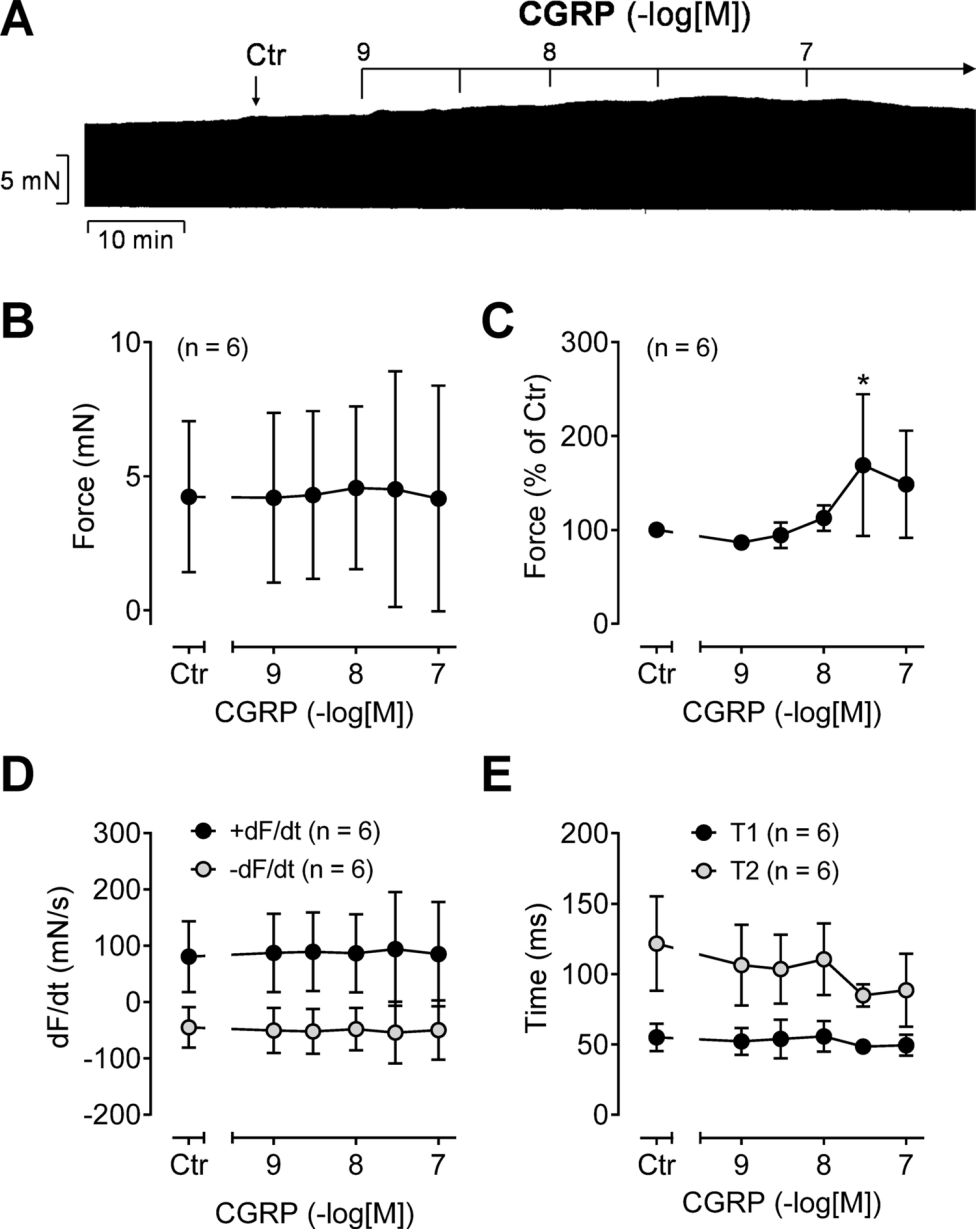
Positive inotropic effect of CGRP in human atrial preparations. **A** Original recording of cumulatively applied CGRP starting at 1 nM. The vertical bar indicates the developed force of contraction in millinewtons (mN), while the horizontal bar denotes the elapsed time in minutes (min). **B**–**E** Summarized data of six experiments: **B** force of contraction in mN; **C** force of contraction in % of control (Ctr = pre-drug values); **D** rate of tension development (+dF/dt) and rate of relaxation (-dF/dt) in mN/s; **E** time to peak tension (T1) and time to relaxation (T2) in milliseconds (ms). Numbers in brackets indicate the number of experiments. *p < 0.05 vs. Ctr

In the initial series of experiments, CGRP was cumulatively applied to HAP, resulting in a modest, concentration-dependent increase in the FOC. This PIE to CGRP is shown in an original recording in Fig. 2A. In Fig. 2B to E, several experiments are summarized. Because of the scatter due to the variability of patient samples (Fig. 2B), the PIE of CGRP becomes more discernible when the data are normalized to the control values (= pre-drug values) (Fig. 2C). Moreover, CGRP raised the absolute values of the rate of tension development and the rate of tension relaxation (Fig. 2D), and CGRP shortened the time to peak tension and the time of relaxation (Fig. 2E).

**Fig. 3:**
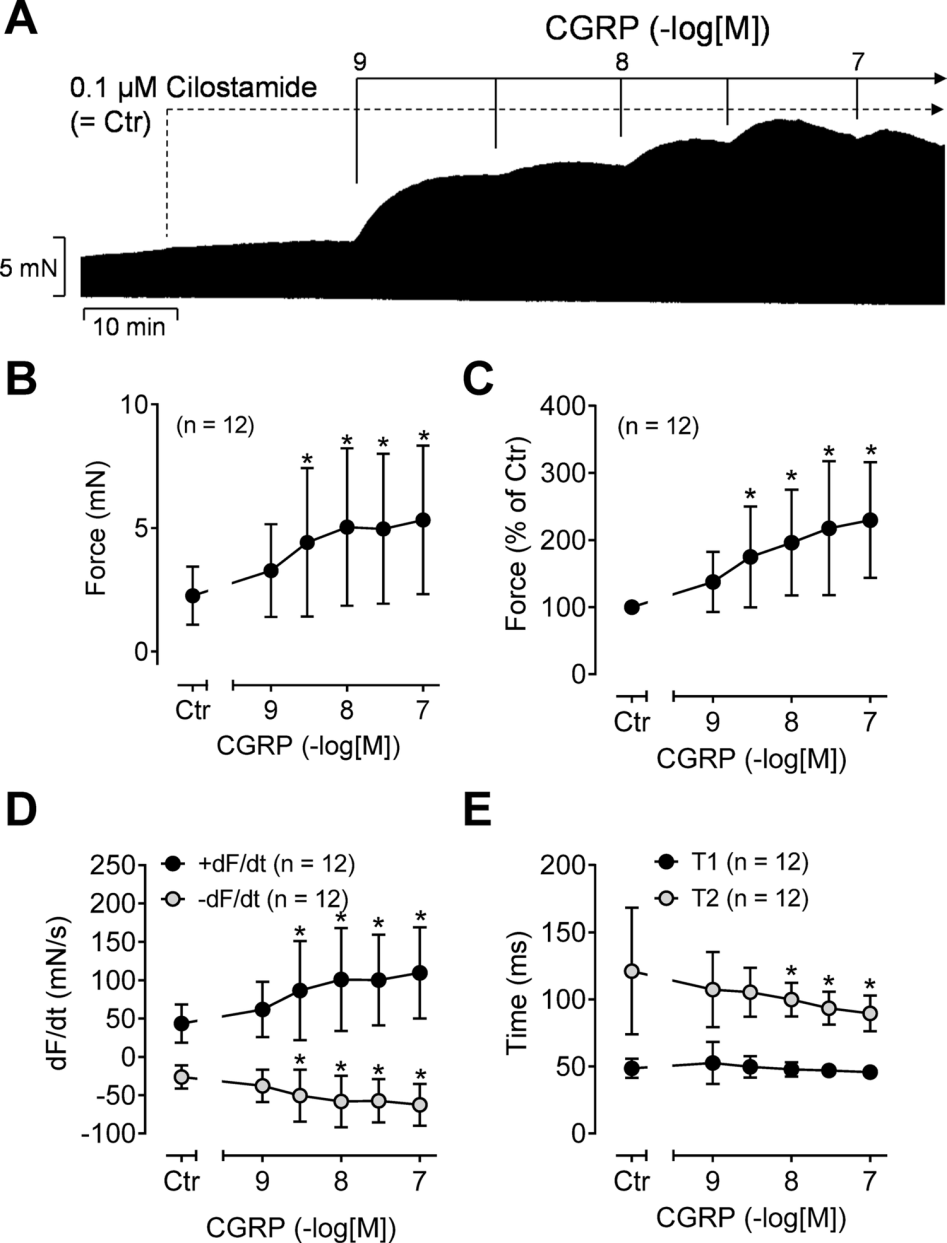
Positive inotropic effect of CGRP in the presence of cilostamide in human atrial preparations. The atrial preparations were pre-stimulated using 0.1 µM of the phosphodiesterase 3 inhibitor cilostamide. **A** Original recording of cumulatively applied CGRP starting at 1 nM in presence of cilostamide. The vertical bar indicates the developed force of contraction in millinewtons (mN), while the horizontal bar denotes the elapsed time in minutes (min). **B**–**E** Summarized data of 12 experiments: **B** force of contraction in mN; **C** force of contraction in % of control (Ctr = force of contraction in presence of cilostamide); **D** rate of tension development (+dF/dt) and rate of relaxation (−dF/dt) in mN/s; **E** time to peak tension (T1) and time to relaxation (T2) in milliseconds (ms). Numbers in brackets indicate the number of experiments. *p < 0.05 vs. Ctr

Secondly, a putative interaction of CGRP and the phosphodiesterase 3 inhibitor cilostamide (Fig. 1) was studied in HAP. A concentration of cilostamide was used that did raise the force of contraction in HAP only slightly, so that a PIE of subsequently applied positive inotropic substances such as CGRP should remain visible (Rayo-Abella et al. 2023). Typical recordings for the effect of CGRP in the presence of cilostamide are depicted in Fig. 3A. In Fig. 3B to E, the experiments are summarized. The PIE of CGRP is significantly more pronounced in the presence of cilostamide than in its absence (compare Figs. 2 and 3). This applies to the absolute FOC values (Fig. 3B) and to the FOC normalized to the control (= effect of cilostamide) (Fig. 3C). In summary, cilostamide shifted the PIE of CGRP to lower concentrations (pEC_50_ = 8.7 ± 0.5 versus 7.7 ± 0.2 without cilostamide, *p* < 0.05) and increased the efficacy. Accordingly, CGRP led to an increase of the rate of tension development and the rate of tension relaxation (Fig. 3D), and CGRP shortened the time to peak tension and the time of relaxation (Fig. 3E).

**Fig. 4:**
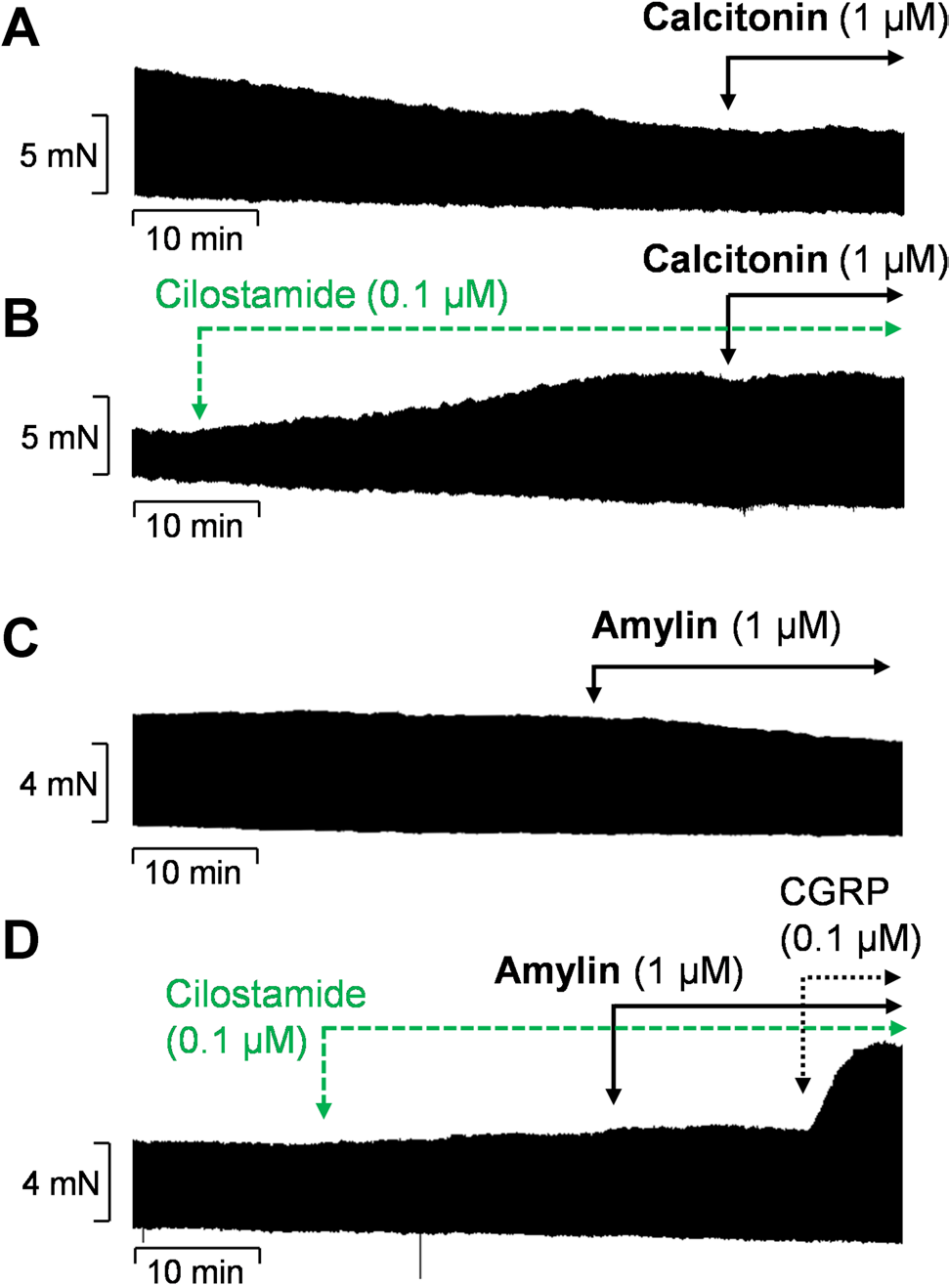
Missing effects of calcitonin and amylin in human atrial preparations. Original recordings of force of contraction demonstrate the lack of inotropic effects of **A** 1 µM calcitonin alone or **B** in the presence of 0.1 µM cilostamide and of **C** 1 µM amylin alone or **D** in the presence of 0.1 µM cilostamide. For comparison, 0.1 µM CGRP was applied in **D** to demonstrate the principle responsiveness to CGRP. The vertical bars indicate the developed force of contraction in millinewtons (mN), while the horizontal bars denote the elapsed time in minutes (min)

In order to exclude the possibility that calcitonin receptors or amylin receptors are involved in the effects of CGRP, calcitonin and amylin were also examined for inotropic activity in HAP. In contrast to CGRP, 1 µM calcitonin did not increase FOC in HAP, when given alone or in the presence of 0.1 µM cilostamide (Fig. 4A, B). A similar outcome was observed with 1 µM amylin, which did not increase the FOC in HAP (Fig. 4C).

**Fig. 5:**
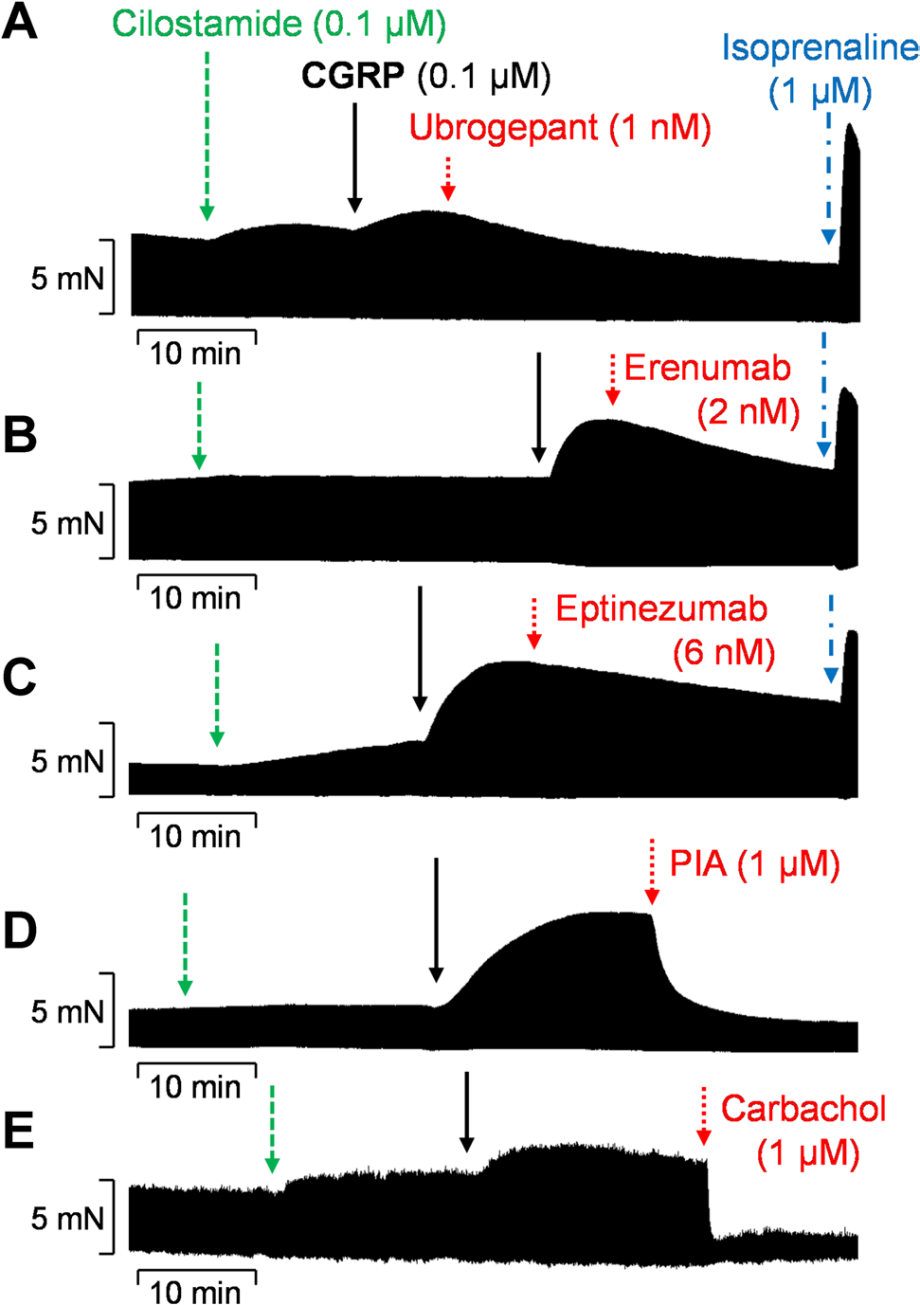
Original recordings of the effects of antagonists on CGRP-induced force of contraction in human atrial preparations. The atrial preparations were pre-stimulated using 0.1 µM of the phosphodiesterase 3 inhibitor cilostamide (green). Subsequently, 0.1 µM CGRP was applied to increase force of contraction followed by **A** 1 nM ubrogepant, **B** 2 nM erenumab, **C** 6 nM eptinezumab, **D** 1 µM PIA, and **E** 1 µM carbachol. All antagonists or negative inotropic compounds are red-colored. In **A**–**C**, 1 µM isoprenaline (blue) was applied at the end of the experiment to demonstrate the maximum capacity to increase the FOC via G_s_ protein–coupled receptors. The vertical bars indicate the developed force of contraction in millinewtons (mN), while the horizontal bars denote the elapsed time in minutes (min)

**Fig. 6:**
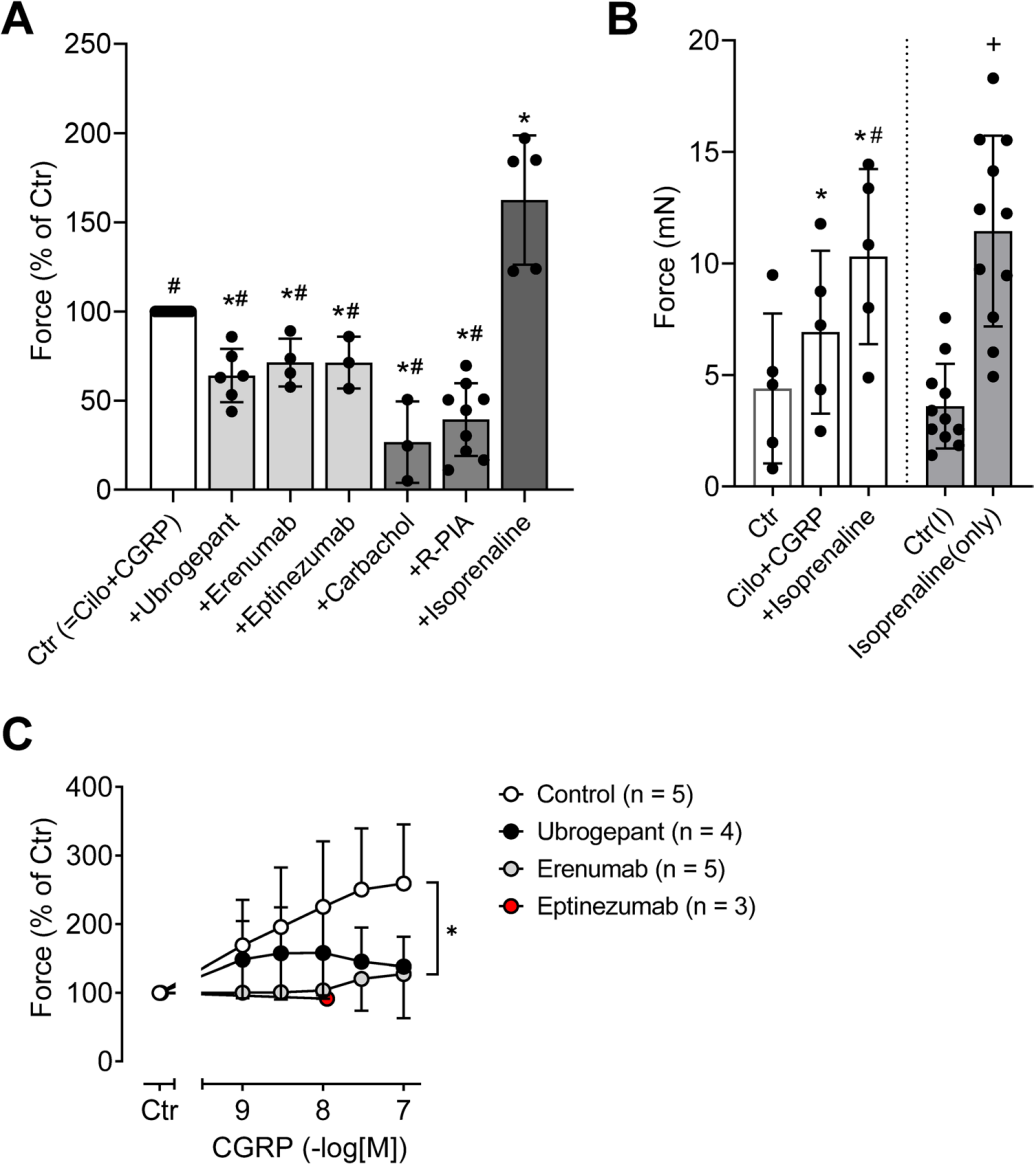
Effects of ubrogepant, erenumab and eptinezumab on CGRP-induced force of contraction in human atrial preparations. **A** Force of contraction (FOC) normalized to the control values (Ctr) that are the effects of 0.1 µM cilostamide plus 0.1 µM CGRP. Ubrogepant (1 nM, *n* = 6), erenumab (2 nM, *n* = 4), and eptinezumab (6 nM, *n* = 3) decreased the FOC to approx. 70%, whereas carbachol (1 µM, *n* = 3) decreased the FOC to 25% and PIA (1 µM, *n* = 9) to 39%. For comparison, 1 µM isoprenaline (*n* = 5) increased the FOC to 162% in the presence of cilostamide, CGRP, and a CGRP or CGRP receptor antagonist. *p < 0.05 vs. Ctr, ^#^*p* < 0.05 vs. isoprenaline. **B** For comparison, the efficacies of 0.1 µM CGRP in the presence of 0.1 µM cilostamide (Cilo) and plus 1 µM isoprenaline (*n* = 5), as well as 1 µM isoprenaline alone (*n* = 11), are shown. Ctr and Ctr(I), corresponding pre-drug values as controls. *p < 0.05 vs. Ctr, ^#^*p* < 0.05 vs. Cilo+CGRP, ^+^*p* < 0.05 vs. Ctr(I). **C** Concentration response curves to CGRP were performed in the presence of both cilostamide (0.1 µM) and an antagonist (either ubrogepant (1 nM) or erenumab (2 nM) or eptinezumab (6 nM)). The positive inotropic effect of CGRP was prevented by all three antagonists tested (*p < 0.05). Data were normalized to cilostamide plus an antagonist (= Ctr). For the control curve, only cilostamide was applied before the addition of CGRP. Numbers in brackets indicate the number of experiments

Finally, the effects of the CGRP receptor antagonists erenumab or ubrogepant as well as the CGRP antagonist eptinezumab were examined (Fig. 5). To this end, concentration response curves to CGRP were performed in the presence of both cilostamide and an antagonist (either ubrogepant or erenumab or eptinezumab). The results of these experiments are demonstrated in Fig. 6, which have been normalized to the control values that are the effects of cilostamide plus an antagonist. The PIE of CGRP was prevented by all three antagonists (Fig. 6C). Consequently, when the antagonists were applied after the highest concentration of CGRP (= 100 nM), the FOC could be reduced by all three antagonists (Figs. 5 and 6A). A post hoc power analysis revealed a statistical power (1-β) of >0.99 for ubrogepant, 0.47 for erenumab, and 0.4 for eptinezumab. For comparison, the interaction with G_i_ protein–coupled receptors was studied because the PIE of other cAMP increasing agents, like isoprenaline, is attenuated by muscarinic M_2_ or adenosine A_1_ receptor stimulation in HAP (Böhm et al. 1986; Schwarz et al. 2024b; Schwarz et al. 2024a). Hence, cilostamide and 100 nM CGRP was applied first. This increased the FOC. Subsequently, carbachol or PIA was applied to stimulate M_2_ or A_1_ receptors, respectively, which reduced the FOC (Figs. 5 and 6A). It should be noted that the negative inotropic effect of carbachol is more pronounced than the negative inotropic effect of PIA (Fig. 5), which is usually explained by a higher receptor density of M_2_ receptors compared to A_1_ receptors (Böhm et al. 1996). To demonstrate the maximum capacity to increase the FOC in HAP via G_s_ protein–coupled receptors, isoprenaline was also applied in several preparations (Figs. 5 and 6). Furthermore, Fig. 6B demonstrates two findings. First, isoprenaline is more efficient than CGRP in the presence of cilostamide to increase the FOC. Second, the β-adrenergic response remained unchanged at the end of the experiments when compared with the effect of isoprenaline alone.

## Discussion

The main objective of the present work was to investigate the potential cardiac effects of the novel anti-migraine drugs ubrogepant, erenumab, and eptinezumab. Indeed, all three investigated drugs were able to reduce CGRP-stimulated contractility in human atrial preparations.

It was known before that CGRP increased the FOC in HAP (Sigrist et al. 1986; Du et al. 1994). These effects were classified as CGRP receptor-mediated because they were antagonized by the CGRP-antagonist CGRP(8–37) in HAP (Saetrum Opgaard et al. 2000). It is also known that the effects of some agonists only become visible after pre-stimulation of the signaling pathway. This has been shown, for example, for the inotropic effects of serotonin in the human ventricle, which was only detectable after inhibition of the cAMP-degrading phosphodiesterases (Kaumann and Levy 2006). However, to the best of our knowledge, it is a novel finding that a PDE inhibitor (here: cilostamide) augmented the PIE of CGRP in HAP. This effect of cilostamide argues against the possibility that CGRP stimulates PDE activity as reported for glucagon in the heart (review: (Neumann et al. 2023)). In contrast, these data support the accepted view that cAMP mediated the PIE of CGRP in the HAP (Sigrist et al. 1986). Another new finding is that the PIE of CGRP is not mediated by calcitonin receptors. We assume that this is the case because calcitonin at a concentration of 1 µM does not increase FOC in HAP. This is consistent with a negative inotropic effect of calcitonin in canine atrial preparations (Chiba and Himori 1977).

Furthermore, it was noteworthy that the action of CGRP in the HAP can be inhibited at different steps of the signal transduction (Fig. 1): one strategy to mitigate the action of CGRP on the CGRP receptor involves the administration of an antibody against CGRP (eptinezumab). Alternatively, a small organic molecule (ubrogepant) or a truncated peptide (CGRP(7–38)) are available to selectively inhibit the stimulation of the CGRP receptor by CGRP. Finally, an antibody that targets the function of the human CGRP receptor (erenumab) can also inhibit the PIE of CGRP in the HAP.

The PIE of CGRP can be partly explained by an increase in the current through the L-type calcium ion channel (LTCC), or by an inhibition of potassium ion currents and a resulting prolongation of the action potential (frog: (Ono et al. 1989), guinea pig atrial cardiomyocytes: (Nakajima et al. 1991)). The PIE of CGRP in guinea pig atrial preparations was antagonized by acetylcholine, and therefore, we studied here carbachol (Nakajima et al. 1991). CGRP shortened the relaxation time and increased the relaxation rate in HAP, probably in part through increasing the protein kinase A-dependent and Ca^2+^-calmodulin kinase-dependent phosphorylation of phospholamban, as illustrated in Fig. 1. The phosphorylated phospholamban would lose its inhibitory effect on SERCA, resulting in an enhanced reuptake of Ca^2+^ into the sarcoplasmic reticulum, which would finally lead to enhanced relaxation. In guinea pig left atria, the effect of CGRP on FOC was potentiated by 3-isobutyl-1-methyl-xanthine (IBMX), a nonselective phosphodiesterase inhibitor, and attenuated by adenosine (Ishikawa et al. 1988). The findings in guinea pigs are largely consistent with our findings in HAP: in our study, the effect of CGRP on FOC was potentiated by the PDE inhibitor cilostamide and attenuated by the A_1_ adenosine receptor agonist PIA. IBMX also potentiated the CGRP-induced increase in cAMP content and in the beating rate in rat neonatal ventricular cardiomyocytes (Fisher et al. 1988). Therefore, we translated these findings from guinea pig atria and rat neonatal cardiomyocytes to HAP, and indeed, we found that the effects of CGRP were enhanced in the presence of a selective PDE inhibitor cilostamide. Furthermore, the PIE of CGRP in HAP was reduced by PIA, an A_1_ adenosine receptor agonist (Schwarz et al. 2024a).

In a human cardiomyocyte cell line (a tumor cell line expressing only calcitonin receptor-like receptors and RAMP1), CGRP increased the cAMP level, calcium ion transients, nitric oxide formation, and phosphorylation of the extracellular signal-regulated kinases ERK1/2 (Clark et al. 2021). However, these data are difficult to put into a physiological context because these human cardiomyocytes showed proliferation and did not contract (Clark et al. 2021).

It has been argued that all currently available CGRP receptor antagonists are also antagonists at amylin receptors (Russo and Hay 2023). Therefore, we might have inadvertently also antagonized the effects of CGRP at amylin receptors, where CGRP is also a weak agonist. However, we consider this as an unlikely limitation of this study. In fact, we could not detect a PIE of amylin in HAP. This finding aligns with previous studies that also failed to detect a PIE of amylin in HAP (Saetrum Opgaard et al. 2000). Thus, we are confident to have studied an action of CGRP at CGRP receptors and not at amylin receptors.

The maximum plasma concentration after 100-mg ubrogepant per os has been reported as 378 ng/ml (approx. 700 nM (Boinpally and Lu 2022)). Depending on the dosage used, plasma levels of erenumab range between 10 and 100 ng/ml (approx. 0.7–7 nM) (Vu et al. 2017). Eptinezumab had a peak plasma concentration of 25–83 µg/ml (approx. 175–580 nM (Li et al. 2023) in patients depending on the dosage. Consequently, the findings of this study are well within the therapeutic range of these medications, thereby substantiating their clinical relevance. In addition, the CGRP concentrations used in this study are close to the range of CGRP plasma concentrations in humans. Although CGRP concentrations show a high inter-individual variability, the mean plasma concentration of CGRP was about 300 pg/ml, i.e., approx. 80 pM, with maximum values of about 0.5 nM in some individuals (Messlinger et al. 2021; Eggertsen et al. 2024). The mRNA for the calcitonin receptor-like receptor, RAMP1, RAMP2, and RAMP3, has been detected in the right atrium and left ventricle of humans (Saetrum Opgaard et al. 2000). In addition, antibodies have been used to detect the calcitonin receptor-like receptor in the human heart, mainly in endothelial cells, but also in human ventricular cardiomyocytes (Hagner et al. 2002). Hence, biochemically, the CGRP receptor is present in the human heart.

Finally, some limitations of the study have to be mentioned. Despite the potential of the novel anti-migraine drugs to decrease the inotropic effects of CGRP, most patients will probably not notice any cardiac effects, as the physiological CGRP concentrations are probably not sufficient to induce a positive inotropic effect without pre-stimulation. This may also be the reason why no cardiac side effects have been reported to date with the novel anti-migraine drugs. Moreover, it could be argued that it would be very helpful to compare our data with data from non-diseased specimens. However, access to non-diseased control hearts is not available at our university hospital. Consequently, this comparison could not be done in the present study. Another point that could not be addressed in principle by our study design are possible chronic effects of CGRP antagonism, such as atrial tissue remodeling or desensitization of the receptor. However, such effects would be important to know, as CGRP antagonists are intended to be used as a preventive therapy for migraine. Such studies need to be carried out in the future on a larger patient population.

In summary, the present study provides evidence that the novel anti-migraine drugs that interfere with the action of CGRP abolish the positive inotropic effect of CGRP in the human atrium.

## Data Availability

The data of this study are available from the corresponding author upon reasonable request.
